# Violence detection explanation via semantic roles embeddings

**DOI:** 10.1186/s12911-020-01237-4

**Published:** 2020-10-15

**Authors:** Enrico Mensa, Davide Colla, Marco Dalmasso, Marco Giustini, Carlo Mamo, Alessio Pitidis, Daniele P. Radicioni

**Affiliations:** 1 Department of Computer Science, University of Turin, Corso Svizzera 185, Turin, 10149 Italy; 2 Servizio sovrazonale di Epidemiologia dell’ASL TO3 della Regione Piemonte, Via Sabaudia 164, Grugliasco (TO), 10095 Italy; 3 Reparto Epidemiologia ambientale e sociale Dipartimento Ambiente e Salute (DAMSA) Istituto Superiore di Sanità, Viale Regina Elena, 299, Roma, 00161 Italy; 4 Data Analysis Services, B2C Innovation Inc. - Digital Services, Corso Magenta 69/A, Milan, PO Box 20123 Italy

**Keywords:** XAI, Explanation, Text categorization, Categorization explanation, Word embeddings, Semantic frames, Slot filling, Event extraction, Violent event tracking

## Abstract

**Background:**

Emergency room reports pose specific challenges to natural language processing techniques. In this setting, violence episodes on women, elderly and children are often under-reported. Categorizing textual descriptions as containing violence-related injuries (V) *vs*. non-violence-related injuries (NV) is thus a relevant task to the ends of devising alerting mechanisms to track (and prevent) violence episodes.

**Methods:**

We present ViDeS (so dubbed after Violence Detection System), a system to detect episodes of violence from narrative texts in emergency room reports. It employs a deep neural network for categorizing textual ER reports data, and complements such output by making explicit which elements corroborate the interpretation of the record as reporting about violence-related injuries. To these ends we designed a novel hybrid technique for filling semantic frames that employs distributed representations of terms herein, along with syntactic and semantic information. The system has been validated on real data annotated with two sorts of information: about the presence vs. absence of violence-related injuries, and about some semantic roles that can be interpreted as major cues for violent episodes, such as the agent that committed violence, the victim, the body district involved, etc.. The employed dataset contains over 150K records annotated with class (V,NV) information, and 200 records with finer-grained information on the aforementioned semantic roles.

**Results:**

We used data coming from an Italian branch of the EU-Injury Database (EU-IDB) project, compiled by hospital staff. Categorization figures approach full precision and recall for negative cases and.97 precision and.94 recall on positive cases. As regards as the recognition of semantic roles, we recorded an accuracy varying from.28 to.90 according to the semantic roles involved. Moreover, the system allowed unveiling annotation errors committed by hospital staff.

**Conclusions:**

Explaining systems’ results, so to make their output more comprehensible and convincing, is today necessary for AI systems. Our proposal is to combine distributed and symbolic (frame-like) representations as a possible answer to such pressing request for interpretability. Although presently focused on the medical domain, the proposed methodology is general and, in principle, it can be extended to further application areas and categorization tasks.

## Introduction

Explanation is acknowledged to be an epistemologically relevant process [1] and a precious feature to build robust and informative systems. It is a matter of fact that artificial explanation has a long tradition in the AI field, and some areas such as case-based reasoning seem to be intrinsically connected to explanatory needs [2, 3]. In machine learning, decision trees [4] and sparse linear models [5] are popular examples of techniques that produce interpretable models. Also in the AI field, some sort of lexical resources have been employed to assist in the construction of the explanation of semantic similarity ratings between word pairs [6, 7]. Many sorts of explanation can be drawn, responding to diverse needs underlying the general aim at providing more transparency to algorithms and systems. For example, the role of explanation in AI systems and its relevance w.r.t. systems accountability is debated in the EU General Data Protection Regulation [8, 9]. On a different side, the tight relation between automatic explanation and trust has been individuated in many contexts as a central issue (think, e.g., to the role of explanation in the field of information security), in its interplay with ethical and sociological issues [10]. Besides, together with the impetuous surge of work on explainable AI, some attempts have been carried out at investigating what constitutes a good explanation, and how the contributions from different disciplines such as psychology and cognitive science can enrich the quality of the explanations being provided by systems [11].

Areas where intelligent systems and agents are currently deployed are as different as personal assistants, logistics, scientific research, law and health care. While in some cases (e.g., some kinds of chatbots) users are not interested in explanations, for sensitive tasks involving “critical infrastructures and affecting human well-being or health, it is crucial to limit the possibility of improper, non-robust, and unsafe decisions and actions” [12]. One chief motivation for building explainable AI systems is thus the need to check systems behavior, to ensure that systems perform as expected. This need has become particularly relevant in the last few years, that have witnessed the spread of deep learning based neural networks, in that these are featured by strong predictive power, at the expense of the interpretability of their output [13, 14]. In this work we investigate one such critical application domain: the categorization of electronic medical records (EMR) data, where an Information Extraction approach has been devised to complement the output of the deep neural network performing the categorization step.

In particular, our system is aimed at categorizing textual descriptions from Emergency Room Reports (ERRs) as containing violence-related injuries vs. non-violence-related injuries, to the ends of devising alerting mechanisms to track violence episodes. The early detection of violence in general, and specifically against women, elderly and childhood is a serious concern for our societies. However, interestingly enough, such phenomena are to date underestimated and not even fully recorded in statistics. Let us consider, for example, that violence against women is seldom reported from its inception due to many reasons, such as the fact that this sort of violence is performed by family members or acquaintances [15, 16]. Likewise, and due to similar reasons, according to the recommendations by Centers for Disease Control and Prevention (CDC), violence on children is largely acknowledged to be under-reported [17]. Additionally, hospital staff may have practical difficulties in properly annotating violence episodes (e.g., complex user interfaces, or lack of time to fully describe the medical history of patients), so that violence and its effects are to date not fully grasped. This determines the necessity to devise automatic systems to automatically detect violence in electronic medical records (EMR) data, so to allow timely intervention and design policies to contrast the phenomenon of violence. From a technical viewpoint, a desideratum would be that of building a classifier to individuate EMRs containing descriptions of violent events in the medical history along with its effects in the physical examination. In order to generate an explanation of the obtained categorization, one would also be able to make explicit the more relevant elements associated to events: by whom the violence was exercised, in what ways, what trauma was produced on the victim, which are the involved body parts, when and where the event has occurred.

This is the focus of the work: we face the problem of extracting meaningful pieces of information to the ends of justifying the categorization performed by the system. We present the VIDES system, so dubbed after ‘VIOLENCE DETECTION SYSTEM’: the designed approach provides violence events with a formal characterization in terms of semantic frames [18]. Additionally, the control strategy devised to fill the frame slots employs a hybrid strategy exploiting distributed word representations together with morphological (on part-of-speech tags) and semantic (on super-senses) information. Experimental results on an annotated dataset containing real ER data are reported and discussed in depth.

## Related work

The closely related task of frame identification has been addressed by [19]: in this work distributed representations of predicates and their syntactic context were exploited, paired with a general purpose set of word embeddings. Our work differs from the mentioned approach, in that we do not make use of syntactic information (since our input is very noisy, which would completely undermine parsing accuracy and reliability). Additionally, we retrain our embeddings on a set of EMR data, to acquire specific descriptors (we are concerned with a very specific application domain, that of first aid medical records) for the Italian language; and finally we are concerned with a more restricted task, that is extracting the fillers for the slots from a single frame, the violence frame.

As regards as acquiring distributed representations to describe verb dependents and semantic frames, word embeddings have been employed also to investigate cross-language misalignment, such as related to polysemy, syntactic valency (i.e., the number of dependent arguments of verbs), and lexicalization [20]. In particular, the authors of the cited work build different embeddings for a given frame, one for each language of interest. Since such embeddings lie in the same semantic space, this approach is used to automatically measure the cross-lingual similarity of language-specific frames to the ends of investigating the possibility of frame transfers across languages. Frame-based approaches have been also adopted, paired to deep syntactic analysis, to elaborate documents from the legal domain through a template-filling approach [21, 22].

Word embeddings have been used also to perform semantic role labeling (SRL); this task is to discover the relations between predicate and its arguments, so it basically amounts to discovering “who” did “what” to “whom”, “when”, “where”, and “how”. This line of research was started in [23], where the distributions over verb-object co-occurrence clusters were used to improve coverage in argument classification. The work by [24] proposes a distributional approach for acquiring a semi-supervised model of argument classification preferences, that is used to reduce the complexity of the employed grammatical features in combination with a distributional representation of lexical features. Additionally, in [25] a selectional preference model has been proposed providing a single additional feature to classify potential arguments based on distributional similarity. The neural network architecture described in [26] relies on the intuition of embedding dependency structures, and jointly learns embeddings for dependency paths and feature combination. The work by [27] proposes to tackle the SRL task by assigning semantic roles through a feedforward network that uses a convolution function over windows of words; interestingly enough, this system does not make use of syntactic information.

With respect to this line of research using word embeddings to perform the SRL task, we face a slightly different problem. First, we are not really concerned with SRL: we are interested in a variant of SRL, where we need to extract salient information (to generate an explanation) associated to a single semantic frame (describing violent events). Additionally, different from the surveyed approaches, our input texts are very challenging and cannot undergo a standard parsing process, as almost any sentence contains typos, acronyms, domain-specific (at times, hospital-specific) abbreviations, and clauses well-formedness

is mostly violated. Such features prevented from designing a suitable sequence of preprocessing steps, and our system deals with all mentioned phenomena without performing rewriting of the input text. This implies that our system substantially differs from those concerned with the SRL task. In fact, most SRL modules perform two main steps, argument identification and argument classification, with the former basically grounded on syntactic parsing, and the latter requiring additional semantic knowledge to solve the task. Instead, our approach puts together word embeddings, supersense tags, and simple part-of-speech (PoS) filtering techniques to the ends of collecting enough information to explain why an Emergency Room Report describes a violence event.

## The system

**Fig. 1:**
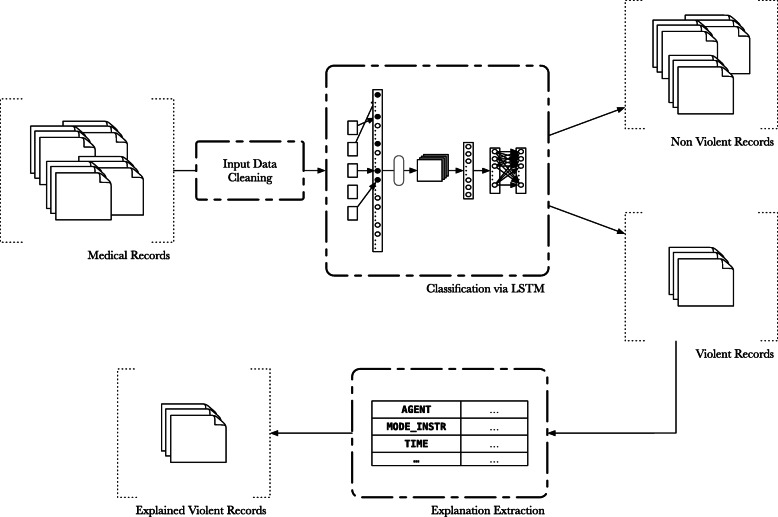
The system outline. A complete outline of the VIDES system. The medical records initially undergo a cleaning step, they are then categorized into violent and non violent ones; subsequently records deemed to contain violence-related injuries are selected for further processing, in order to obtain an explanation of such categorization

Before describing in full details the software modules implementing the VIDES system, we provide a high level overview, also depicted in Fig. 1.

First element of our pipeline is the data cleaning step, necessary to deal with this sort of input, that for several reasons appears as intrinsically noisy [28, 29]. Then the categorization module performs the classification of the medical records in order to assign a label (V if violence related injuries were detected, NV otherwise), determining whether the record exhibits traits of violence or not. Records that have been categorized as containing injuries resulting from violent episodes are further processed to extract salient information on the violence episode available in the text. Finally, the categorized records are returned, enriched with the most salient elements describing the violence event. To these ends, we devised a hybrid approach that exploits distributional, semantic and syntactic information.

The input to the VIDES system is compliant to an EU-level standard, as defined within the Injury DataBase (EU-IDB) framework [30]. Each record in the dataset contains various types of information, such as the age and gender of the patient, the type of trauma, the medical report describing the trauma in detail and a narrative report describing the events that led the patient to the emergency room. The schema is however differently implemented in various countries of the Union [31]; therefore, in order to possibly extend the system to handle further countries’ medical records, we decided to use as few record fields as possible. In particular, in our experimentation we only consider the narrative report, since this is the field providing the most relevant information to build the explanation.

### Input data cleaning

Emergency room reports are often very noisy, to such an extent that most of the records contain at least one word which cannot be found in a standard dictionary. This fact has several causes. Personnel compiling the entries is often in a hurry, which may explain misspellings and typos; also, in this kind of text the usage of abbreviations is by far more frequent than in general language; additionally, as it mostly happens in technical settings, domain-specific terms are also recurrent. As a result, this mixture of errors, abbreviations, acronyms and domain-specific terms makes dealing with such documents a challenge for artificial systems.

**Fig. 2:**
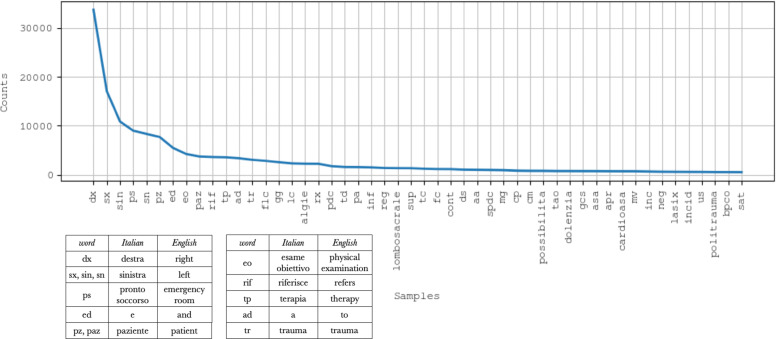
Distribution of out-of-vocabulary terms. Distribution of the 50 most frequent acronyms, abbreviations and misspelled terms in the dataset

The *input data cleaning* phase fixes multiple spaces and punctuation errors by applying regular expressions [32], and then it applies a rewriting technique: based on a medical dictionary, the most frequent abbreviations and acronyms are expanded while also correcting recurring typos. For instance, the word ‘destra’ —‘right’ in English— is rarely used in the corpus, while its abbreviation ‘dx’ is widely adopted. Purpose of this phase is then to replace each occurrence of ‘dx’ with the corresponding ‘destra’. The medical dictionary contains 248 entries, and it has been manually compiled by medical experts, focusing on the most frequent abbreviations, acronyms and typos found in the corpus. Figure 2 illustrates the distribution of the 50 most frequent abbreviations, acronyms and typos found in the dataset. The curve represents a very steep Zipfian distribution, therefore, despite its simplicity, the medical dictionary is very effective. Specifically, over the 150K records a total of 178,111 substitutions are applied during the input data cleaning phase. As expected, abbreviations and acronyms are used consistently, while typos appear to be more diverse and varied through the dataset. The design of a more complete and robust solution, also able to take into consideration a wider variety of typos, is currently under active development and it will be addressed in future work.

### Neural model to track violence related injuries

**Fig. 3:**
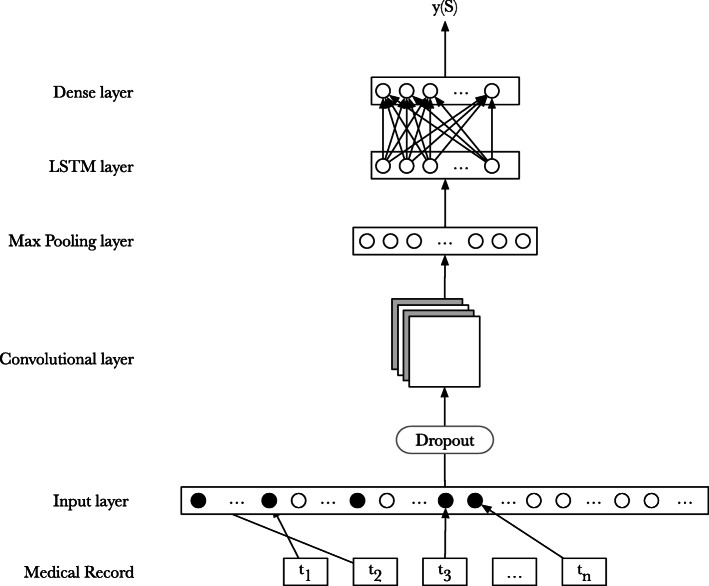
The neural network architecture. The neural architecture employed for the categorization task

As regards as the categorization of the medical records, a neural model has been devised to discriminate among violent and non-violent entries. Such architecture is illustrated in Fig. 3.

Input to the model is the rewritten text contained in the ER record; such text is first tokenized and then mapped onto a numerical vectorial representation. The mapping from terms to vectors 〈*term*,*numerical* *id*〉 was acquired from the considered dataset. More specifically, the weight matrix has been initialized with 300*-d* fastText word vectors trained on the whole dataset by adopting the SkipGram architecture [33]. The input layer is then connected to a single dimension convolutional layer, composed of 64 filters; the kernel consists of 5 units and adopts the Rectified Linear Unit (ReLU) activation function. A dropout rate of 20% was set between the input layer and the convolutional layer in order to reduce the overfitting of the model. A max pooling layer with a window size set to 4 units was adopted to reduce the dimension of the input, and is followed by an LSTM layer built with 100 memory units. Finally, a fully connected layer —adopting the sigmoid activation function— is used to predict the class of the medical record: V for violent episodes, and NV for non-violent episodes. In this setting the role of the convolutional layer is twofold: *i)* to learn abstract features coming from medical reports; and *ii)* to reduce the training time. The training phase employs Adam stochastic optimization [34] and binary cross entropy loss function.

The output of this module is the categorized ER record.

### Building explanations by frame elements embedding

The second module is fed with the entries that were recognized as violent by the network, and is intended to extract information relevant to describe a violence episode: this amounts to filling the slots (that can be thought of as object fields) of a violence frame.

The *frame* is a popular representational device in the fields of lexical semantics and knowledge representation [35]; a frame is a “system of concepts related in such a way that to understand any one of them you have to understand the whole structure in which it fits; when one of the things in such a structure is introduced into a text, or into a conversation, all of the others are automatically made available” [36, p. 373]. One chief assumption of this work is that violence related injuries can be recognized not by starting from scattered words, but rather when the core elements of that ‘violence frame’ are extracted. Individuating such elements (when available in the ER report) is of the utmost importance to the ends of explaining and deepening the binary output provided by the neural categorization model. Explaining the categorization involves filling the semantic components of the violence frame.

The violence frame contains the most salient information ordinarily associated to violence events, and it is thus defined as follows.

- Agent: The agent performing the violence. Example phrases may be ‘known person’, ‘husband’, ‘wife’, etc.;
- Mode-Instrument: The mode or the instrument adopted while performing the violence. Examples of this field are ‘punch’, ‘aggression’, ‘knife’, etc.;
- Time: Temporal information regarding when the violence occurred. Examples are ‘evening’, ‘night’, ‘today’, etc.;
- Location: The physical place in which the violence took place. Examples are ‘home’, ‘workplace’, ‘bus station’, etc.;
- Body-Part: Body part harmed by the violent act. Examples are ‘arm’, ‘head’, etc.;
- Lesion-Type: Type of injury produced by the violent act. Examples are ‘fracture’, ‘contusion’, ‘trauma’, etc..

All of the mentioned fields may have zero or multiple fillers, depending on the content of the considered entry. Also, attached to each field *f* we have two lists: *PoS*_*f*_ and *SST*_*f*_, indicating the part-of-speech (PoS) tags and SuperSense tags (SST) that a filler for *f* can assume.

PoS tags are grammatical categories associated to words, such as noun, verb, pronoun, preposition, adverb, conjunction, adjective, and article. Knowing the PoS associated to a given word is a noun or a verb provides relevant information about likely neighboring words (e.g., in English nouns are preceded by determiners and adjectives, verbs by nouns and adverbs, etc.) and syntactic structure. PoS tagging is thus an important enabling task for natural language processing. PoS tagging is not directly mapping words onto their PoSs, because a given word can be possibly tagged with different PoSs, based on the different contexts where it occurs [37]. Also, PoS taggers are featured by high accuracy when both training and test data are drawn from the same corpus, while performances typically drop in front of words unseen in the training set [38]. In domain-specific applications, this effect is limited, so that PoS tags can be considered as providing reliable information.

Whereas PoS tagging is concerned with the grammatical level of linguistic processing, super-sense tagging (SST) targets the semantic category of words in their context of occurrence, performing a basic form of word sense disambiguation [39]. Super-senses can be thought of as a set of semantic categories; although in principle different sets of such tags can be adopted, the tagset from the online dictionary of WordNet [40] is customarily employed, containing overall 41 semantic categories, 26 super-senses for nouns and 15 for verbs. Super-senses are actually the roots of as many trees partitioning noun and verb senses in WordNet. Each super-sense represents a broad semantic category, such as SST.NOUN_PERSON or SST.NOUN_LOCATION, which can be exploited to either accept or rule out candidates for our frame slots.

The two sets *PoS*_*f*_ and *SST*_*f*_ are used to match the semantic needs (intended as the set of semantic limitations and requirements) of each frame slot *f* with the morphological and semantic information available in the actual input text. For example, the AGENT field can only be filled by a SST.NOUN_PERSON or POS.NOUN. Table 1 reports the two sets designed to rule out possibly inappropriate arguments.

**Table 1:** Compatibility table illustrating the allowed PoSs and SSTs for each explanation frame field

**Field**	**Part Of Speech**	**SuperSense Tags**
Agent	Noun	Noun_Person
Mode-Instrument	Noun	Noun_Object
		Noun_Artifact
		Noun_State
		Noun_Substance
		Noun_Feeling, Noun_Act
Time	Noun	Noun_Time
Location	Noun	Noun_Location
Body-Part	Noun	Noun_Body
Lesion-Type	Noun, Adjective	Noun_State, Adj_all
		Noun_Phenomenon

In the extraction step, after a basic preprocessing consisting of the punctuation removal, we identify locutions (hereafter referred to as *extended tokens*) whose elements are common multi-word expressions found in the dataset that should be processed as a whole (e.g., *known_person*). Finally, the sentences are tokenized.

We then proceed to the construction of a candidate set of fillers for each field: given a field *f*, we initialize its set of candidates *C* to all terms in the input record. Then, we prune from *C* all terms whose PoS or SST is not compatible with the needs of the semantic field *f*. More precisely, for each term *t*∈*C* we retrieve its PoS and its most frequent SSTs. Namely, PoS tagging is computed through the Tint parser, an Italian porting [41] of the Neural Network Dependency Parser [42]. Supersense tags are computed by accessing WordNet and retrieving the most frequent sense, among all senses possibly underlying a given input term. Although this may seem a rough simplification, the most frequent sense is experimentally acknowledged as a competitive baseline [39], and used as a core feature in more sophisticated SST systems [43], ensuring limited computation time and effort. Given the rather narrow semantic domain for the present application, thus featuring a reduced problem space, we opted for this simple heuristics. The term *t* is retained only if its PoS is included in *PoS*_*f*_ and at least one of its SSTs is included in *SST*_*f*_. Extended tokens bypass this process, and they are all included as candidates by default.

Once we have filtered *C* so that it contains only terms that are allowed as fillers for *f*, we rank them by leveraging the fastText embeddings acquired through the first module. Namely, for each field we build a synthetic vector by averaging the most frequent terms that can possibly act as fillers. In this way we build a vector $\hat {f}$ containing a synthetic description for each semantic role *f* of the frame; all candidate words *c*∈*C* are then ranked based on their distance from $\hat {f}$. The last of the algorithm consists in building the final answer provided by the system. Here, we apply two strategies: *1)* all candidates *c* in the ranking whose similarity with a given field vector is lower than a certain threshold are discarded (this parameter has been optimized and set to.5); and *2)* if, after the pruning, a term is still a candidate for more than one field, it is assigned to the closest field.

**Fig. 4:**
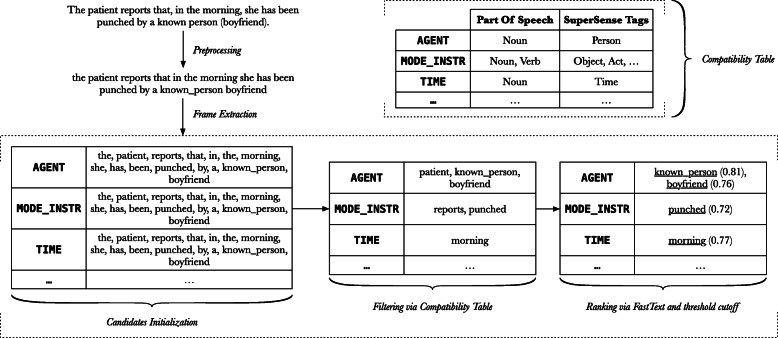
Example of frame extraction for a sentence. The sentence extracted from the medical record is initially preprocessed, and then given in input to the frame extraction process. Every word in the sentence is considered as a potential candidate for each role. Candidates are then filtered and ranked, and the top scoring one is selected in order to obtain the final filler for each frame element

Figure 4 provides an example translated from Italian into English to illustrate the whole process. We consider a record that has already been recognized by the Neural module as containing a violence related injury. The sentence herein is extracted from the medical record and preprocessed. Every word in the sentence is considered as a potential candidate for each frame field; i.e., each field is initially assigned to the whole set of candidates. These sets of candidates are then pruned by taking into consideration the semantic needs of each frame element (Table 1). Finally, the best candidate for each semantic role is chosen by exploiting the similarity calculated via fastText vectors.

**Running Example.** In order to recap the pipeline of the VIDES system, let us consider the following example sentence taken from an ER record, and its processing all throughout the described pipeline:


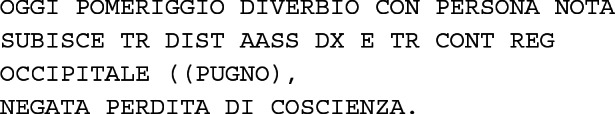


The sentence can be translated into ‘This afternoon brawl with a known person, suffers from tr dist aass dx reg occipital ((fist), loss of consciousness denied’ (abbreviations were not translated, and the mismatch of the brackets was left unaltered). The cleaning step allows us to fix the punctuation, to perform the lowercase conversion of the sentence, and most importantly to rewrite some of the abbreviations. Namely, ‘tr’ is replaced with ‘trauma’ (‘injury’), ‘dx’ is replaced with ‘destro’ (‘right’), ‘cont’ is replaced with ‘contusivo’ (‘blunt’), ‘reg’ is replaced with ‘regione’ (‘region’, ‘body district’). However, since ‘aass’ (standing for ‘arti superiori’, ‘upper limbs’) is not present in the medical dictionary, it is not rewritten thus remaining unchanged.

The resulting sentence —‘this afternoon brawl with a known person suffers from distorting trauma aass right and blunt trauma in occipital region (fist), loss of consciousness denied’— is then used as input to the neural network, which performs its own preprocessing by removing the punctuation and tokenizing the text. The system correctly categorizes the record as a violent one.

Finally, since the record has been recognized as V, the explanation module is executed. It initially recognizes extended words, such as *known_person*, and it then computes the best candidate for each field. The result of the extraction process is the frame below. For each semantic role in the frame, we report the filler (the top ranked term) along with the associated cosine similarity score, compactly expressing its compatibility with the event frame:


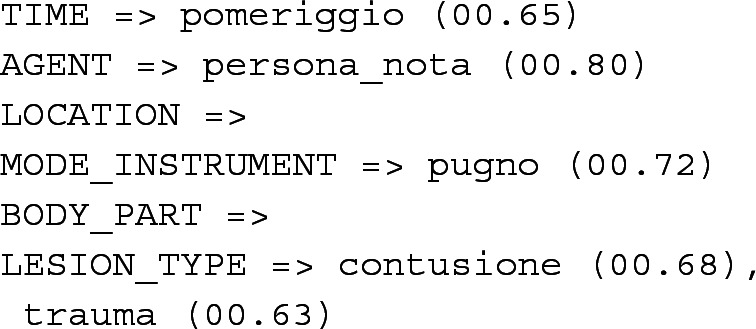


The final result is: TIME (afternoon), AGENT (known person), MODE-INSTRUMENT (fist) and LESION-TYPE (contusion and trauma). Fillers are successfully extracted and assigned to the appropriate frame element, with the only exception of the body part. The body parts involved in the violent act are the upper limbs and the occipital region. The first one cannot be correctly extracted since it was not rewritten from its abbreviation ‘aass’, while the similarity between the embeddings of ‘occipital’ and BODY-PART does not reach the required threshold.

## Evaluation

### Dataset and procedure

The data used in the experimentation are real-world emergency room reports (ERRs) collected in Italian Hospitals, and then made available by the Italian National Institute of Health in the frame of the SINIACA project [44]. The SINIACA project (so dubbed after ‘Sistema Informativo Nazionale sugli Incidenti in Ambiente di Civile Abitazione’, National Information System on Accidents in Civil Housing Environment) is the Italian branch of the European Injury Database (EU-IDB) [30], an EU-wide surveillance system concerned with accidents, collecting data from hospital emergency department patients according to EU recommendation. SINIACA is a data collection on home injuries, based on a sample of hospital emergency departments, in implementation of the recommendation of the Council of the European Union no. C 164/2007/01 on injury prevention and safety promotion.

For our experimentation we have used 153,823 records from the SINIACA-IDB, which were originally annotated by hospital staff as containing injuries descending from either violent (V in the following) or non violent acts (NV in the following). The dataset is very unbalanced, as it contains 5,168 records that were tagged as violent, while the remaining 148,655 (96.64*%*) were labeled as not caused by violent acts. The dataset has been randomly split into 2 equal parts: the former one was used for training and parameters tuning (80:20 the ratio between training and validation set, respectively); the rest was used as our test set. The dataset is indeed very unbalanced, since records annotated as V amount to 3.36*%* of the whole data. The two partitions —for training and testing, respectively— were designed so to preserve the same distribution in both training and test set.

As regards as the two modules of our system, we have then recorded the classification accuracy obtained on half the dataset (about 76,900 records) by the classifier implemented through the neural network. As regards as the evaluation of the explanation generated, we annotated 200 randomly sampled records among those returned as violent (V) at categorization time. We were concerned with detecting descriptions of violence related injuries, so we did not use a finer-grained annotation schema, e.g. discriminating among violence against women, the elderly and minors. Each such record was associated to a frame, whose fields were filled with the information available in the text document. Provided that each frame contains 6 fields, overall 1200 slots were annotated: in 729 cases a filler was annotated from the accompanying text, whilst in 471 cases no value could be set. More specifically, the available information associated widely varied across the slots, as follows: AGENT was filled in the 60% of cases; MODE-INSTRUMENT was filled in the 97% of cases; TIME was filled in the 23.5*%* of cases; LOCATION was filled in the 8% of cases; BODY-PART was filled in the 89.5*%* of cases; LESION-TYPE was filled in the 86.5*%* of cases. Since multiple annotations were allowed (according to the information available in the input text), we recorded overall 5.53 fillers annotated for each record (e.g., the lesion type can be both ‘trauma’ and ‘wound’; the involved body part can be ‘shoulder’, ‘leg’ and ‘arm’): more specifically AGENT was filled on average with 0.66 elements; MODE-INSTRUMENT was filled on average with 1.44 elements; TIME was filled on average with 0.28 elements; LOCATION was filled on average with 0.09 elements; BODY-PART was filled on average with 1.77 elements; LESION-TYPE was filled on average with 1.29 elements.

Such annotated data was set as our ground truth annotation, against which the frame computed by the explanation module was compared.

### Results

#### Categorization results

The categorization is aimed at detecting medical records containing violence by employing the neural model. The training and validation of the model was performed on 76,911 records randomly sampled from the whole dataset; the test involved as many items.

The results of the categorization step are reported in Table 2: we obtained a 99% F1 score for the non-violence class. The F1 score for the V class is 86%. The neural model identified 2,291 entries as violence (V) cases; regarding the V class, the true positives amount to 2,073 out of 2,584 items, thereby yielding a.92 precision and a.80 recall. Overall, 218 false positives were detected (i.e., such data was labeled as V by the system, but annotated as NV by hospital staff).

**Table 2:** Precision, Recall and F1 scores for violence (V) and non-violence (NV) classes on the test set

**Class**	**P**	**R**	**F1**
midrule NV	.99	1.0	.99
V	.92	.80	.86

The precision of the categorization module on the V class ensures that the explanation module (taking as input the records labeled as V at categorization time) is mostly executed on records describing injuries related to violence events. The set of records labeled by the neural model as V has then been used to assess the accuracy of the explanation module, concerned with extracting the relevant information to fill the violence frame slots.

#### Frame elements extraction results

The frame element extraction was validated by comparing the extracted elements against human annotations earlier illustrated in the “Dataset and procedure” section. In order to evaluate the quality of the extracted fillers we have analyzed each field separately. The following metrics (standard in Information Retrieval tasks) were recorded to assess the output of the system:

- Mean Average Precision (MAP): the mean of the average precision obtained over all dataset, where the average precision is the precision of each element given as result;
- Success at 1 (S@1): the percentage of cases in which the first value was correct;
- Success at 5 (S@5): the percentage of cases in which among the first five values the correct value was returned.
- Recall at 5 (R@5): how many of the correct values were returned among the first five values.

Additionally, we developed a baseline against which we compared the output of the proposed approach. The baseline adopts the same pre-processing as the main algorithm, with the difference that it only employs semantic similarity to rank the results; the similarity threshold was also preserved, but no PoS tag/SST filtering was employed. The detailed results are provided in Table 3.

**Table 3:** Results for the explanation algorithm along with the baseline

**Run**	**Field**	**MAP**	**S@1**	**S@5**	**R@5**
Baseline	Agent	.12	.12	.14	.13
	Mode-Instrument	.24	.22	.29	.27
	Time	.73	.74	.74	.73
	Location	.50	.51	.51	.50
	Body-Part	.15	.18	.26	.18
	Lesion-Type	.30	.36	.37	.31
Main algorithm	Agent	.58	.59	.60	.58
	Mode-Instrument	.28	.31	.32	.29
	Time	.80	.82	.82	.80
	Location	.90	.90	.90	.90
	Body-Part	.49	.57	.57	.49
	Lesion-Type	.40	.45	.47	.41

The whole control strategy always favorably compares to the baseline, thereby showing that PoS tagging and SST information are helpful to extract the information to fill the frame slots.

### Discussion

As regards as the neural network module, the VIDES system showed satisfactory accuracy in categorizing both NV records (.99 F1 score) and V records (.86 F1 score).

The output of this module can thus be considered as reliable, to such an extent that it has been used to check and to correct, although in supervised fashion, the information collected in real-word, hospital records. In fact, a closer inspection of the false positives revealed that in 65% of cases (that is, 141 out of 218 false positives) the system had predicted V, mistakenly annotated by the hospital staff as NV. For example, the record with text ‘[...] the patient reports that he had been beaten by known person, all over his body but especially on the right shoulder [...]’ had been annotated as NV, while the VIDES system had predicted it as a violent case (V). In such cases the annotation is wrong. After manually correcting such errors, the precision obtained by the VIDES system raises to 97% for the V class. The updated figures are reported in Table 4, where we observe a consistent +5*%* improvement in the precision w.r.t. results in Table 2.

**Table 4:** Precision, Recall and F1 scores for violence (V) and non-violence (NV) classes on the test set, after correction of the mistakenly annotated false positives

**Class**	**P**	**R**	**F1**
NV	.99	1.0	.99
V	.97	.81	.88

Similarly, we have further investigated the false negative cases. The system classifies 74,621 entries as NV, 74,110 of which are annotated as NV (true negatives) and 511 as V (false negatives). A closer inspection of these 511 records reveals that most records (namely 367, amounting to 72% of cases) are wrongly annotated. In most cases (211 out of 367) no description is available, and also in the remaining cases too little information is present, so that neither domain experts would be able to discriminate between V and NV cases. Different from the aforementioned cases regarding false positives, here we did not overwrite the experts’ annotation which we use as ground truth, since we had absolutely no cues to determine whether an item was V or NV. Records with lacking or insufficient information to make a decision deserve further inspection, by resorting to their annotators, and by focusing specifically on this phenomenon. This will be done in future work. Presently, we simply dropped poorly informative records, thereby obtaining a consistent improvement in the recall of the VIDES system, as illustrated in Table 5, whose results can be compared to those in the previous tables to complete the assessment on the categorization task.

**Table 5:** Precision, Recall and F1 scores for violence (V) and non-violence (NV) classes on the test set, after correction of the mistakenly annotated false positives and deletion of false negatives

**Class**	**P**	**R**	**F1**
NV	1.0	1.0	1.0
V	.97	.94	.95

The task of extracting the relevant pieces of information to fill the violence frame confirms to be a challenging and stimulating one. Different degrees of difficulty feature the recognition of the relevant frame elements. TIME and LOCATION of the violence event were individuated to a greater extent than other elements, such as the MODE-INSTRUMENT, LESION-TYPE and BODY-PART. As regards as such fields, we note that on average more information was available (e.g., MODE-INSTRUMENT was filled in 97% of cases, with 1.445 fillers, on average, over the 200 considered records), that may have been detrimental to the exact identification of such elements.

A closer inspection at the errors in the generation of the explanation may be beneficial for future improvements and for similar applications grounded on the adoption of distributed word representations paired with the filling of semantic frames.

Some errors in the recognition of the AGENT originate from the fact that further persons can be mentioned in the ER report (e.g., in a sentence like ‘the father reports that the patient was punched by her husband’). In such cases neither PoS nor SST information are helpful in filtering out the father as the author of the violence: this sort of errors should be dealt with through syntactic parsers (at least to individuate the dependent clause ‘the patient was punched by her husband’), thus permitting to rule out ‘father’ as the agent.

Further errors stem from the SST filtering step: in some cases even such basic disambiguation performed through supersense tags fails, thereby undermining the filtering step. This determines a too crowded set of candidates, and these elements are not properly ranked in the subsequent stage. Errors in the SST are in principle equally distributed across all classes, but their impact is worse on frame elements having more general semantic types as admissible candidates, such as MODE-INSTRUMENT and, of course, for terms with a higher degree of polysemy. The primary role of SST information is also confirmed by the comparison between the baseline and the fully fledged VIDES system.

Many errors were caused by typos: even the trivial lack of a space between two words may prevent the tokenizer from correctly recognizing the terms involved in the linguistic expression, and tools could be adopted that have been designed for the interactive correction and semantic annotation, also with special focus on narrative clinical reports [45, 46].

Additionally, one desideratum would be individuating multiword expressions such as ‘neck of the bottle’ or ‘lacerated bruised wound’ that need to be handled as a whole (and that, conversely, cannot be dealt with in a token by token mode) [47]. Unfortunately, in the considered domain and for the considered text excerpts, standard approaches such as mwetoolkit [48] are frequently mislead to such an extent that their adoption does not ensure substantial processing advantage.

To improve the performance in the task of semantic frame extraction, it would be thus crucial to benefit from reliable syntactic (either dependency or constituency parsing [49, 50]) information, which unfortunately could not be attained, due to the presence of frequent disfluencies, ungrammatical structures and out-of-vocabulary (OOV) tokens. Also, a richer representation of the frame elements could be obtained by employing knowledge graph embedding techniques [51, 52], that can be combined with predictive models [53], although these cannot alleviate the issues stemming from the poor quality of the input. In facts, ER reports are conceived as short reports for hospital insiders, rather than as a complete, fully explicit, grammatically and syntactically correct form of communication.

This is definitely what makes them intriguing and worth research efforts, like many other forms of contemporary communication, featured by similar grammatical and syntactical traits such as, e.g., social media communication [54], and some forms of spoken language involving ill-formed spontaneous spoken language and under-specified grammars [55].

## Conclusions

In this article we have presented the VIDES system, aimed at providing the categorization computed through a neural-network based classifier with explanations. In particular, we have considered the task of categorizing emergency room reports, by focusing on those containing violence events. We have illustrated the motivations underlying this kind of application: contrasting violence, by promptly tracking violent episodes as they are reported in the ER setting. On a purely scientific viewpoint, we have illustrated some of the challenges inherent to performing information extraction tasks when dealing with this type of language.

The input to the VIDES system is composed by text documents that, as illustrated, can be hardly elaborated with standard (e.g., syntactic parsing) NLP techniques due to many typos, abbreviations, acronyms, and so forth. As mentioned, we have cast the present task to a particular sort of Semantic Role Labeling, where the system has to fill the slots describing a violence event, that has been previously fed to the neural model. In order to explain why a record was labeled as containing a violence-related injury, the VIDES system performs a hybrid step of information extraction by employing word embeddings, supersense tags, and PoS filtering techniques.

To the best of our knowledge, no attempt has been proposed yet to tackle this task by exploiting a synthetic (vectorial) representation for each semantic slot. Although improvements can be drawn, this approach showed to obtain encouraging results, especially for some kinds of information. It would be interesting to investigate to what extent our approach generalizes to further applications in the medical domain and to further domains, as well. Although some components of the proposed pipeline rely on domain-specific knowledge tailored to the application needs (in particular the dictionary and the vector representation of the event frame), in principle the presented methodology may be applied to different settings in order to build explanations of various sorts of output of neural models.

## Data Availability

The data that support the findings of this study are available from the National Institute of Health (Istituto Superiore di Sanità, ISS) but restrictions apply to the availability of these data, which were used under license for the current study, and so are not publicly available. Data are however available from the authors upon reasonable request, and with permission of ISS.

## Abbreviations

EMR: electronic medical record
ERR: emergency room report
V: class whose records contain description of a violence related injury
NV: class whose records do not contain description of a violence related injury
CDC: Centers for Disease Control and Prevention
ViDeS: Violence Detection System
SRL: semantic role labeling
PoS: part of speech
ER: emergency room
ReLU: rectified linear unit
LSTM: long short-term memory
SST: super sense tag
SINIACA: ‘Sistema Informativo Nazionale sugli Incidentiin Ambiente di Civile Abitazione’, National Information System on Accidents in Civil Housing Environment
IDB: injury database
MAP: mean average precision
S@1: success in the first element of the output
S@5: success in the first five elements of the output
R@5: recall of the first five elements of the output
OOV: out of vocabulary.
